# Genetic and Chemical Diversity of Edible Mushroom *Pleurotus* Species

**DOI:** 10.1155/2022/6068185

**Published:** 2022-01-15

**Authors:** Pei Lin, Zheng-Fei Yan, MooChang Kook, Chang-Tian Li, Tae-Hoo Yi

**Affiliations:** ^1^Engineering Research Center of Edible and Medicinal Fungi, Ministry of Education, Jilin Agricultural University, Changchun, 130118 Jilin Province, China; ^2^School of Pharmaceutical Sciences, Jiangnan University, 1800 Lihu 12 Avenue, Wuxi, 21422 Jiangsu Province, China; ^3^State Key Laboratory of Food Science and Technology, Jiangnan University, 1800 Lihu Avenue, Wuxi, 21422 Jiangsu Province, China; ^4^Department of Food & Nutrition, Baewha Women's University, Seoul, Republic of Korea; ^5^College of Life Science, Kyung Hee University, Yongin-si, Gyeonggi-do 17104, Republic of Korea

## Abstract

The genus *Pleurotus* is one of the most widely cultivated and edible mushrooms with various cultivators. Three molecular characteristics were used to evaluate the genetic diversity of 132 tested samples. Phylogenetic analysis showed five clades for tested samples of the genus *Pleurotus* by the combined ITS and LSU sequences with strong bootstraps and Bayesian posterior probability supports. A total of 94 polymorphic fragments ranging from 10 to 100 bp were observed by using an intersimple sequence repeat (ISSR) marker. The DNA fragment pattern showed that *P. ostreatus* cultivator (strain P9) was clearly distinguished from wild strain based on their clear banding profiles produced. DNA GC content of the genus *Pleurotus* varied from 55.6 mol% to 43.3 mol%. Their chemical composition was also determined, including sugar, amino acid, polar lipid, mycolic acid, quinone, and fatty acid, which presented some high homogeneity. Most of the tested samples contained mycolic acid; glucose and arabinose as the main sugars; aspartic acid, arginine, lysine, tyrosine, and alanine as the main amino acids; and C_16:0_, C_18:0_, C_18:2_*cis*-9,12, *anteiso*-C_14:0_, and summed feature 8 as the main fatty acids. In addition, their polar lipid profiles were investigated for the first time, which significantly varied among *Pleurotus* species. The genus *Pleurotus* contained menaquinone-6 as the sole respiratory quinone, which showed a significant difference with that of its closely related genera. These results of this study demonstrated that the combined method above could efficiently differentiate each *Pleurotus* species and thus be considered an efficient tool for surveying the genetic diversity of the genus *Pleurotus*.

## 1. Introduction

The species of the genus *Pleurotus* are among the most cultivated and consumed edible mushrooms in the world [1]. Currently, several *Pleurotus* species can be grown commercially to high yields, such as *Pleurotus ostreatus* and *Pleurotus eryngii*. Their production has exceeded 15 million tons each year, which was ranked second following *Lentinula edodes* [2]. In addition to their nutritional value, the genus *Pleurotus* is a natural source of prebiotics and antioxidants [3] and is thus of great interest to the food industry. Also, the genus *Pleurotus* showed a broad spectrum of biological activities [4, 5], including antitumor, antidiabetic, and antibacterial activities.

Currently, the morphological characteristics have laid down the foundation for *Pleurotus* identification. Nonetheless, the characteristics of *Pleurotus* cultivars are unstable and dependent on the environmental conditions, which are too limited in terms of accurate identification [6]. With the development of molecular technology, highly conserved regions in ribosomal DNA are served as the reference points to investigate genetic and evolutionary relationships within species for providing unequivocal species delimitation rule [7, 8], such as 18S, internal transcribed spacers (ITS), and large subunit rDNA (LSU) (Figure S1).

Besides, molecular fingerprinting is also an efficient tool in investigating the genetic diversities for the purpose of breeding programs because of its properties such as independence of environmental parameters and the high levels of detectable polymorphism [9], including simple sequence repeat (SSR), random amplified polymorphic DNA (RAPD), amplified fragment length polymorphism (AFLP), sequence-related amplified polymorphism (SRAP), and intersimple sequence repeat (ISSR) markers. Among them, an obvious advantage of the ISSR marker is that it has no sequence data for primer construction and is randomly distributed throughout the genome. ISSR primers (AG, GA, and (GATA)n repeats) that are anchored to genomic DNA making targeted simple sequence repeats could generate a wide array of amplification products, which provide sufficient information in determining genetic relationships [10]. DNA GC content as an important molecular characteristic has been widely used in taxonomic descriptions of species and genera [11]. However, only a few species of *Pleurotus* have been investigated to analyze their GC content [12, 13].

Chemical characteristics were provided as supplementary criteria to more accurately describe species, which are being given more and more attention [14]. These approaches were used way before DNA-based methods and are mainly regarded as obsolete or less informative nowadays, yet not necessarily useless. Sugar and amino acid profiles as a complementary tool have been applied to identify varieties of *Gibberella fujikuroi* [15]. Studies on chemical characteristics of the genus *Pleurotus* have made some progress [16, 17], but the study is not wide enough.

In the present study, a total of 132 tested samples were collected from Korean Agricultural Culture Collection (KACC, Korea), National Agrobiodiversity Center (NAAS, Korea), Korean Culture Center of Microorganisms (KCCM, Korea), Culture Center of Microorganisms Jilin Agricultural University (CCMJ, China), and market (Homeplus in Yongton-si of Korea) (Table S1). Subsequently, all tested samples were investigated to analyze their genetic and chemical diversity and to compare the differences between them. This information will promote the efficient identification of the genus *Pleurotus*.

## 2. Materials and Methods

### 2.1. Strains and Cultivation

All strains were cultivated in potato dextrose broth (PDB, BD products, Franklin Lakes, NJ, USA) at 32°C for 2 days as seed culture, and then, the seed culture (3% *v*/*v*) was transferred into 50 mL PDB at 32°C for 10 days. Mycelium was obtained by centrifugation at 10,000 g for 10 min and freeze-dried for further investigation as tested samples.

### 2.2. Phylogenetic Analysis

The genomic DNA from an overnight culture was obtained by using the Qiagen Genomic Tip 500G Kit, following the manufacturer's instructions, except that the lysozyme was replaced with lysostaphin to a final concentration of 200 *μ*g/mL and then was stored at -20°C. ITS and LSU regions were performed by using universal primers (primers ITS4 and ITS1 for ITS, primers LR0R and LR3 for LSU). Then, amplification products were sequenced by Biofact Co. Ltd, (Seoul, Korea). All generated sequences were submitted to GenBank and are listed in Table S1.

The sequences of tested samples together with reference sequences from GenBank (Table S2) were aligned by using BioEdit and ClustalX. Alignment was manually adjusted to allow maximum alignment and to minimize gaps. Maximum parsimony analysis was applied to the combined ITS and LSU sequences. The sequence of *Agaricus bisporus* obtained from GenBank was used as an outgroup. PAUP version was carried out as a phylogenetic tree construction procedure. All characters were equally weighted with gaps as missing data. The phylogenetic tree was estimated by using TBR branch swapping and 1,000 random sequence additions. Max-trees were set to 5,000, branches of zero length were collapsed, and all parsimonious trees were saved. The robustness of clades was tested by using a bootstrap (BT) analysis with 1,000 replicates. Then, several factors of Maximum Parsimonious Tree (MPT) were calculated, such as homoplasy index (HI), descriptive tree statistics tree length (TL), rescaled consistency index (RC), retention index (RI), and consistency index (CI). The best evolution for each data set was determined for Bayesian inference (BY) by using MrModeltest 2.3. Bayesian inference was performed by using MrBayes 3.2.3 with a general time reversible (GTR) model of DNA substitution and a gamma distribution rate variation across sites. Four Markov chains were run for 2 runs from random starting trees for 2 million generations, and trees were sampled every 100 generations by discarding the first one-fourth generations as burn-in [18]. A majority rule consensus tree of all remaining trees was calculated. Branches were considered significantly supported with 80% of maximum parsimony (MP) and 0.95 of Bayesian posterior probabilities (BPP).

### 2.3. Genetic Diversity Analysis

A total of 24 primers were collected and applied in this study (Table 1). Then, primer, annealing temperature (47°C, 50°C, 53°C, 60°C, 65°C, and 70°C), and the number of cycles (10, 15, 20, 25, 30, and 35) were studied. The ISSR amplifications were carried out in a 20 *μ*L reaction volume containing 50 ng of the template DNA, 0.75 *μ*M of the primers, 2.5 mM of MgCl_2_, 0.2 mM of dNTPs (TaKaRa, Japan), and 0.5 U of *Taq* DNA polymerase. The amplification conditions were as follows: an initial denaturation at 94°C for 5 min followed by the corresponding cycles each at 94°C for 30 s, 45 s at the annealing temperature, and 72°C for 90 s, followed by the final extension for 7 min at 72°C. The ISSR amplification products were detected on 1% agarose electrophoresis gels, and the images were captured using the ChemiDoc XR (Bio-Rad, USA). Unambiguous and reproducible bands in successive amplifications were selected for scoring. Each fragment was scored as “1” and “0” for the presence/absence, and a genetic distance matrix from raw data was constructed by using the PHYLIP 1.0 package [19]. Cluster analysis was performed by the unweighted pair group method with arithmetic averaging (UPGMA) [20].

### 2.4. DNA GC Content Analysis

The genomic DNA was obtained by using the Qiagen Genomic Tip 500G Kit and purified by using genomic DNA purification kits (Intron Biotechnology Inc., Seongnam, South Korea). Briefly, the purified DNA was decomposed into nucleotides, and the DNA GC content was determined by the method of Tamaoka and Komagata [21] using reversed-phase high-performance liquid chromatography (RP-HPLC).

### 2.5. Sugar Analysis

Cell wall sugars were extracted, purified, and determined by two-dimensional thin-layer chromatography (2D-TLC) [22]. Sugar was identified by comparing sugar standards. The sugar standards were obtained from Sigma: galactose (Gal), glucose (Glu), rhamnose (Rha), arabinose (Ara), xylose (Xyl), and ribose (Rib).

### 2.6. Amino Acid Analysis

The amino acid profiles of each tested sample were determined by using HPLC analysis. Chromatography conditions were in accordance with the Agilent method [23]. Briefly, an amount equivalent to 2.5 *μ*L of each sample was injected on a Zorbax Eclipse-AAA column (5 *μ*m, 150 × 4.6 mm) (Agilent), at 40°C, with detection at 338 nm. Mobile phase A was 40 mM NaH_2_PO_4_, adjusted to pH 7.8 with NaOH, while mobile phase B was acetonitrile/methanol/water (45/45/10 *v*/*v*/*v*). The separation was obtained at a flow rate of 2 mL/min with a gradient program that allowed for 1.9 min at 0% B followed by a 16.3 min step that raised eluent B to 53%. Then, washing at 100% B and equilibration at 0% B were performed in a total analysis time of 26 min. The amino acid was identified by comparing calibration chromatogram established by 10 known amino acids, such as arginine (Arg), alanine (Ala), aspartic acid (Asp), valine (Val), cysteine (Cys), glutamic acid (Glc), glycine (Gly), lysine (Lys), threonine (Thr), and tyrosine (Tyr).

### 2.7. Polar Lipid Analysis

The polar lipids were extracted and determined by the 2D-TLC method [24]. And then, various lipids were identified by their different unique staining characteristics corresponding to their chemical structure.

### 2.8. Quinone Analysis

Quinone was extracted and analyzed by HPLC with the Zorbax-ODS column (4.6 × 250 mm) under the following conditions: mobile phase: methanol : diisopropyl ether (3 : 1, *v*/*v*); flow rate: 1 mL/min; detector: photodiode-array detector scanning from 200 to 400 nm; and UV detector at 275 nm for ubiquinones and at 270 nm for menaquinones [25]. Their type was identified by comparing the relative retention times of peaks from standards, such as quinones-8 (Q-8), quinones-9 (Q-9), quinones-10 (Q-10), menaquinones-5 (MK-5), menaquinones-6 (MK-6), menaquinones-7 (MK-7), menaquinones-7(H2) (MK-7(H2)), menaquinones-7(H4) (MK-7(H4)), menaquinones-7(H6) (MK-7(H6)), menaquinones-8 (MK-8), menaquinones-8(H6) (MK-8(H6)), menaquinones-9 (MK-9), and menaquinones-10 (MK-10).

### 2.9. Mycolic Acid Analysis

To investigate the distribution of mycolic acid in the genus *Pleurotus*, mycolic acid was extracted, purified, and analyzed by using TLC with petroleum ether/acetone (95/5 *v*/*v*) as the developing solvents. After air drying, dots were visualized by iodine fumigation [26].

### 2.10. Fatty Acid Analysis

Fatty acid profiles were determined by gas-liquid chromatography and identified by using the Sherlock Microbial Identification System (MIDI) [27]. The relative percentage of each fatty acid was calculated by internal normalization of the chromatographic peak area.

## 3. Results and Discussion

### 3.1. Phylogenetic Analysis

**Figure 1:**
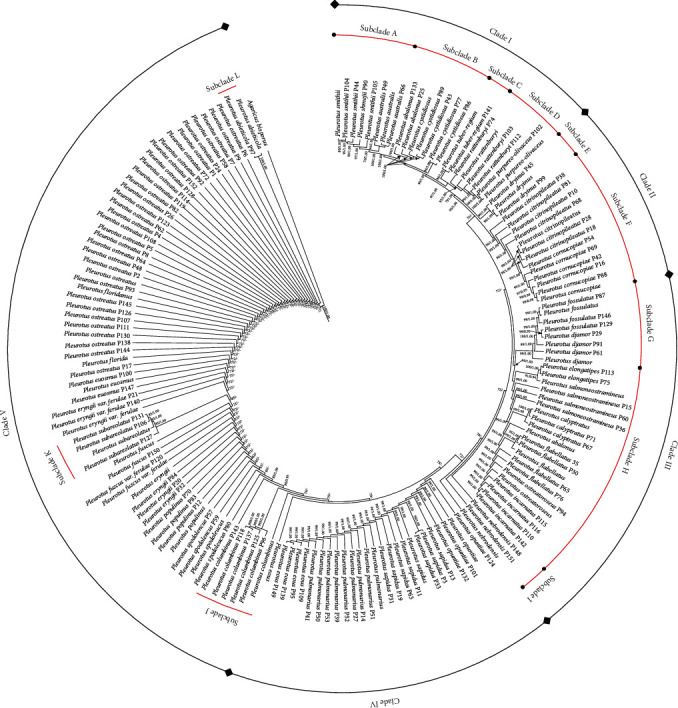
Phylogenetic analysis of species in the genus *Pleurotus.* Strict consensus tree was generated by maximum parsimony based on combined ITS+LSU sequences. Parsimony bootstrap proportions (before the/) higher than 80% and Bayesian posterior probabilities (after the/) more than 0.95 were indicated along branches. The reference sequences of *Pleurotus* (bold) were downloaded from GenBank (Table S2).

In this study, about 100 equally parsimonious trees were produced by maximum parsimony analysis with RI = 0.652, TL = 1132, RC = 0.302, CI = 0.367, and HI = 0.632. The same topology was also obtained by Bayesian analysis with an average standard deviation of split frequencies (0.009). As shown in Figure 1, a well-resolved phylogenetic tree was constructed by using the combined sequences of ITS and LSU.

All tested strains were split into five clades, and most of these clades were recovered by the combined ITS and LSU sequences with strong bootstraps and Bayesian posterior probability supports. Clade I was formed and comprised four subclades, and subclade A was composed of *Pleurotus smithii* and *Pleurotus australis*, indicating that they were closer in a relationship. Subclade B included *Pleurotus abalonus* and *Pleurotus cystidiosus*. Previously reported *P. abalonus* was also considered to be a subspecies of *P. cystidiosus* (*P. cystidiosus* subsp. *abalonus*) [28], which strongly supports results of our study. However, there were significant differences in morphology between them. *P. cystidiosus* exhibited a specific anamorphic stage of the genus *Pleurotus*, which is manifested by the presence of arthroconidia on conidiophores assembled in the coremium macrostructures, suggesting the ability for asexual reproduction independently, as a possible mechanism for effective dispersal of these species in the wild [29]. *P. tuber-regium* was separated as an individual subclade (C) in clade I. *Pleurotus rattenburyi* was identified as novel species by Redhead and Norvell, which was closely related to *Pleurotus purpureo-olivaceus* [30], and they made up subclade D in this study. Clade II was composed of *Pleurotus dryinus*, *Pleurotus citrinopileatus*, and *Pleurotus cornucopiae*. Among them, *P*. *dryinus* was formed as an individual subclade (E). Both *P. citrinopileatus* and *P. cornucopiae* made up subclade F, suggesting a closed relationship between them at the generic level. *P. citrinopileatus* and *P. cornucopiae* were classified as belonging to the same intersterility group, despite the confirmed differences in their ITS sequences [31]. Then, Petersen and Krisai-Greilhuber reconsidered *P. citrinopileatus* species status and define it as *P. cornucopiae* var. *citrinopileatus* [32]. However, since differences between both species are detected not only at the molecular level but also in their morphology, further examinations and additional crosses between representatives of both species are needed to definitively confirm or deny the existence of interspecific reproductive barriers among them. Clade III was composed of *Pleurotus fossulatus*, *Pleurotus djamor*, *Pleurotus elongatipes*, *Pleurotus salmoneostramineus*, *Pleurotus calyptratus*, *Pleurotus flabellatus*, *Pleurotus ostreatoroseus*, *Pleurotus incarnatus*, and *Pleurotus nebrodensis*, which comprised three subclades (G, H, and I). *P. salmoneostramineus* was revised as *P. djamor*, but they are not in the same subclade. Similar results also prevailed in *P. fossulatus* and *P. nebrodensis*. The origin and taxonomic status of *P. flabellatus* were difficult to determine by previous data, which was associated with *P. cornucopiae* [33], or considered an intermediate between the *P. ostreatus* and the *P. eryngii* clades [34]. Previous studies suggested that *P. calyptratus* is a variation within the *P. djamor* [35]. However, both species were divided into two subclusters on the phylogram in this study. Meanwhile, they are also differentiated by their habitat; for example, *P. calyptratus* is distributed in the temperate climate zone, while *P. djamor* is common in a warm tropical climate zone. Their edibility is also different; for example, *P. djamor* has been cultivated commercially and edible. In turn, *P. calyptratus* are hard and are not edible. It was confirmed that *P. calyptratus* and *P. djamor* represent a single species. Most interestingly, the taxonomic position of *P. elongatipes* remained uncertain, which was proposed as *Hypsizygus elongatipes* in Index Fungorum and *P. elongatipes* in MycoBank. In our study, two samples of *P. elongatipes* were gathered together with a 100% bootstrap value and 1.00 Bayesian posterior probability, and it strongly grouped with *P. salmoneostramineus* (91% MP, 0.94 BPP), which has given qualified support to views of MycoBank. *P. nebrodensis* also formed a monophyletic clade by a weak support (less than 80% MP), suggesting that it is slightly distant from other species in clade III. Clade IV included *Pleurotus opuntiae*, *P. sapidus*, *Pleurotus pulmonarius*, and *Pleurotus eous*. Our analysis inferred that *P. opuntiae*, *P. sapidus*, *P. pulmonarius*, and *P. eous* formed a well resolved monophyletic clade with strong support (100% MP, 1.00 BPP). Our study indicated that *P. sapidus* was distinct from *P. cornucopiae* at the genetic level, which differed from the view of Albert et al. [35], suggesting that both represent a single species. Clade V as the largest clade was composed of *Pleurotus columbinus*, *Pleurotus spodoleucus*, *Pleurotus populinus*, *P. eryngii*, *Pleurotus fuscus* var. *ferulae*, *Pleurotus fuscus*, *Pleurotus subareolatus*, *Pleurotus eryngii* var. *ferulae*, *Pleurotus euosmus*, *P. ostreatus*, and *P. abieticola*. Most of them formed a monophyletic clade by a weak support (less than 80% MP). Interestingly, *P. abieticola*, *P. columbinus*, and *P. spodoleucus* formed a well-supported monophyletic subclade with an 85-100% bootstrap value and 1.00 Bayesian post probability, respectively. Previous phylogenetic studies have given rise to various ambiguities in the genus *Pleurotus*. *P. ostreatus*, *P. columbinus*, and *P. cornucopiae* have been in turn associated in the same clade [36] or separated [34]. In our analysis, *P. cornucopiae* on clade III and *P. columbinus* and *P. ostreatus* on the clade V were included in two distinct clades. It was to be noted that the close relationship between *P. columbinus* and *P. ostreatus* has been previously reported [37] and that they have recently been described as sexually compatible species [38]. Sequences of *Pleurotus florida* and *Pleurotus floridanus* obtained from GenBank were gathered together with *P. ostreatus* by a weak support (less than 80% MP). Previous data has been described that *P. floridanus* as the invalid name was revised as *P. ostreatus* [30] and *P. florida* was geographical isolates from the *P. ostreatus* complex, also called *P. ostreatus* [39]. It was suggested that they might have a close genetic relationship, which strongly supported our results.

### 3.2. Genetic Diversity Analysis

A total of 24 primers were applied to investigate genetic diversities of the tested samples by using the ISSR marker (Figure S2). Among them, primers R1 and R3 failed to produce any PCR products and were discarded from further analysis. From the total number of bands obtained and the percentages of polymorphisms of each tested primer, 52 out of 76 bands were considered polymorphic, generating a polymorphism information content (PIC) overall average of 68.4%. The maximum percentage of polymorphism was observed using primer R17 (90.0%), while the lowest percentage was observed using primers R10 and R24 (50.0%). To increase the richness and legibility of fragments, optimal annealing temperature cycle number was detected at 53°C and 25, respectively (Figure S3). The annealing temperature was often higher than Tm, which has more polymorphic fragments than that of other annealing temperatures [10].

**Figure 2:**
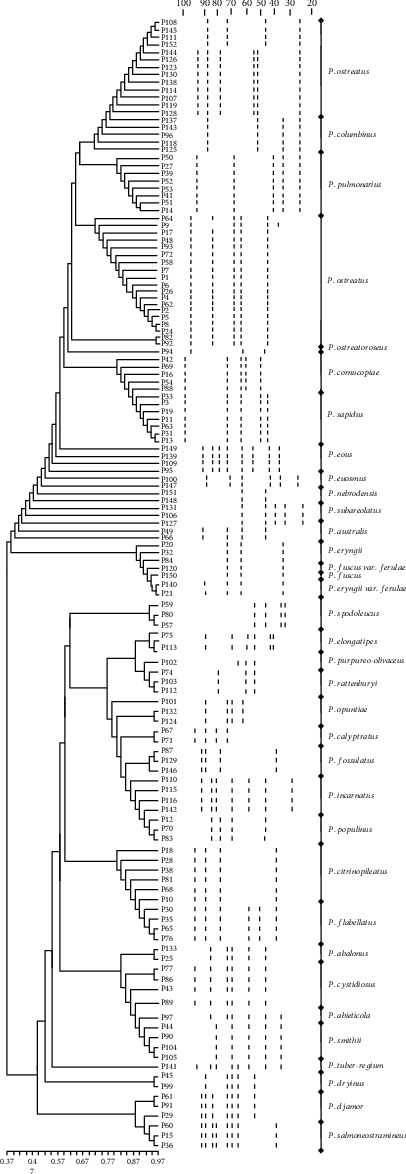
Genetic diversity of species in the genus *Pleurotus* by UPGMA dendrogram. All data were analyzed by using the PHYLIP 1.0 package. Cluster analysis was performed by the unweighted pair group method with arithmetic averaging (UPGMA).

In this study, a total of 94 polymorphic fragments ranging from 10 to 100 bp were observed, which have a higher polymorphism level than those in a previous study [40]. As shown in Figure 2, the average coefficient is 0.67, ranging from 0.37 to 0.97. Based on the UPGMA dendrogram, all tested samples were clustered into several clusters corresponding to their respective taxa; polymorphic fragments were almost consistent with the coefficient of 0.65 at the intraspecies level. A special polymorphic fragment was detected in the *P. ostreatus* cultivator (strain P9), which has significant differences in wild strain. The breeding stage might have a significant impact on the genetic diversity of *P. ostreatus*, which was consistent with a previous report [41]. The ISSR marker was also considered to be a more useful method in distinguishing *P. eryngii* isolates and varieties [42, 43]. The genetic similarity among *P. eryngii*, *P. fuscus*, *P. fuscus* var. *ferulae*, and *P. eryngii* var. *ferulae* ranged from 86% to 96%, indicating the existence of high genetic diversity between them, which was supported by previous studies [42]. *P. eryngii* var. *ferulae* and *P. eryngii* appeared to have a distinct polymorphic fragment, which was consistent with previous studies [44]. Interestingly, 33 strains of *P. ostreatus* were gathered into 2 subclades in the UPGMA dendrogram, which were obviously different from our results by phylogenetic analysis (Figure 1). In the first subclade, the former name of strains was *P. floridanus* (P108, P145, P111, and P152) and *P. florida* (P144, P126, P123, P130, P138, P114, P107, P119, and P128). Our results showed a significant difference polymorphic fragment between *P. floridanus*, *P. florida*, and *P. ostreatus*. It was confirmed that *P. floridanus* and *P. florida* represent a single species, which was in line with the opinion of Gonzalez and Labarère [45].

### 3.3. DNA GC Content Analysis

DNA GC content of the genus *Pleurotus* varied from 43.3 mol% in *P. opuntiae* to 55.6 mol% in *P. fuscus* var. *ferulae*. Meier-Kolthoff et al. confirmed that the threshold value of GC content was 3-5% within species [11]. Exceptions did exist, especially with fungi, which sometimes altered inconsistently with the above-said patterns. A threshold value of GC content was 8% in yeast [46] and 1% in the genus *Trichosporon* [47]. Only a few species of the genus *Pleurotus* have been investigated (Table 2). In addition, GC content of other species of the genus *Pleurotus*, such as *P. nebrodensis*, has not yet been determined despite their importance for different industries. In this study, the range of GC content was slightly different from the values reported previously. This might be caused by incubation conditions of the mycelium, which was supported by Cui et al. [48].

### 3.4. Sugar Analysis

Sugar profiles of all tested samples showed some high homogeneity (Table 3). All of them contained Glu, Ara, and Xyl. Part sugars slightly varied among species, such as Gal, Rib, and Rha. This phenomenon also appeared in that of yeast [49]; for example, Xyl and Ara existed in different yeast species. Our results showed that the types of sugar in tested samples were in agreement with previous results [50]. Currently, sugar might play an important role in food and provide the majority of energy for human being. Meanwhile, Jacob et al. confirmed that *Pleurotus* was low in sugar and became a popular low-calorie food [51].

### 3.5. Amino Acid Analysis

A total of 10 types of amino acid were detected in the tested samples (Table 3), such as Asp, Arg, Lys, Tyr, Ala, Cys, Gly, Thr, Glc, and Val. All of them contained Asp, Tyr, and Ala. Then, other types of amino acid varied with the different species of the genus *Pleurotus*. For example, *P. eryngii* was distinguished from *P. eous* by the presence of Thr. Patil et al. found similar amino acid profiles in *P. ostreatus*, which strongly supports our results [52]. Previous reports also confirmed that cultivation materials of the genus *Pleurotus* have a significant effect on its content of amino acids; for example, *P. citrinopileatus* cultivated on paddy straw and other agrowaste combination has a higher content of amino acids than that on paddy straw alone [53].

### 3.6. Polar Lipid Analysis

Currently, only a few articles described the polar lipid of the genus *Pleurotus*, but most focused on its content and activity [54]. Polar lipid profiles of the genus *Pleurotus* were investigated for the first time in this study (Table 3). Both phosphatidylcholine (PC) and phosphatidyl-N-methylethanolamine (PME) were detected in all tested samples. And other types of polar lipid varied with the different species of the genus *Pleurotus*, such as diphosphatidylglycerol (DPG), phosphatidylglycerol (PG), phosphatidylserine (PS), phosphatidylethanolamine (PE), unidentified phospholipid (PL), unidentified aminolipid (AL), unidentified glycolipid (GL), and unidentified lipid (L). *P. tuber-regium* could be distinguished from others by the absence of DPG. Similar results also appeared in *P. elongatipes*, which was the lack of PE.

### 3.7. Quinone Analysis

So far, there were no correlated publications reporting quinone profiles of the genus *Pleurotus*. This study first investigated quinone profiles of the genus *Pleurotus* (Table 4). Our results showed that all tested samples contain MK-6 as the sole respiratory quinone, which was consistent with previous studies that menaquinone often exists in Gram-positive species with a high GC content [55]. As far as the genus *Pleurotus* is concerned, our results showed that there is no remarkable correlation between quinone types and *Pleurotus* species. Subsequently, closely related genera were also investigated to determine quinone types, such as *Lentinellus*, *Hohenbuehelia*, *Resupinatus*, and *Phyllotopsis*. Results revealed that quinone type is different from that of the genus *Pleurotus* (Table 4), which was used as chemical characteristic in distinguishing the genus *Pleurotus* from its closely related genus. Environmental factors such as nutritional components, oxygen, and temperature are known to affect lipid content and composition in living organisms, including fungi [49].

### 3.8. Mycolic Acid Analysis

Mycolic acid has been characterized in Mycolata taxon [56]. However, there are no previous reports that record mycolic acid distribution in the genus *Pleurotus*. In our studies, the distribution of mycolic acid in the genus *Pleurotus* was detected for the first time. Results showed that mycolic acid was detected in all tested samples, except in *P. tuber-regium*. It was suggested that *P. tuber-regium* could be distinguished from others by using mycolic acid as a key indicator.

### 3.9. Fatty Acid Analysis

As shown in Table 5, a total of 43 types of fatty acid have been detected in the genus *Pleurotus*, including *anteiso*-C_14:0_, C_16:0_, C_18:2_*cis*-9,12, C_18:0_, C_18:3_*cis*-6,12,14, and summed feature 8 (C_18:1_*ω*7c and/or C_18:1_*ω*6c) as the main fatty acids. 16 of 43 types of fatty acid were observed for the first time, including C_9:0_, C_18:1_ 2OH, C_15:1_*ω*8*c*, C_16:1_*ω*5*c*, C_16:1_*ω*7*c* alcohol, C_16:1_*cis*-9 (*ω*7*c*), C_16:1_*cis*-11 (*ω*5*c*), C_17:1_*cis*-10 (*ω*7*c*), C_19:1_*trans*-7 (*ω*9*c*), C_20:1_ N alcohol, C_20:1_*cis*-11 (*ω*9*c*), *iso*-C_14:1_, *iso*-C_15:1_, *iso*-C_17:1_ at 5 (*ω*12*c*), *iso*-C_17:1_ G (*ω*11*c*), and *iso*-C_18:1_. Other types of fatty acid were previously found in the genus *Pleurotus* [16, 57]. The longer uneven chain fatty acids C_21_-C_25_ were not detected in our tested samples. However, it has been detected in *P. djamor* [53]. In most previous data, polyunsaturated fatty acids had over 40% of the total fatty acids, including linoleic acid (C_18:2_*cis*-9/*cis*-12) and a-linolenic acid (C_18:3_*cis*-6/*cis*-12/*cis*-14). In this study, C_18:2_*cis*-9,12 and C_18:3_*cis*-6,12,14 were detected in most tested samples and presented in high amounts compared with other fatty acids.

Table 5 shows the different levels of major and minor fatty acids. *P. columbinus* has the significantly highest proportion of C_16:0_ (100%) as solar fatty acid, which is reported for the first time in the genus *Pleurotus*. It was suggested that a particular species might intrinsically display a higher proportion of a specific fatty acid when compared with others. The application of fatty acid composition data has now extended to studies of physiology, chemotaxonomy, and intrageneric differentiation, as well as human nutrition. But its content in mushrooms is extremely flexible and always influenced by environmental conditions such as media composition, pH, temperature, and growth stage [57].

## 4. Conclusion

Referring to these findings from this study, surveying the genetic variation through phylogenetic analysis, ISSR marker, and GC content could be useful in efficiently differentiating each *Pleurotus* species. The subsequent analysis of their chemical characteristics was consistent with the above results. The combined molecular and chemical analysis could provide a solid and reliable tool for *Pleurotus* classification and taxa delimitation.

## Figures and Tables

**Table 1:** The primers used in this study for ISSR analysis.

Primers	Sequence (5′-3′)	Total bands	Polymorphic bands	PIC (%)
R1	(CT)_7_CC	0	0	0
R2	(AC)_8_C	5	4	80.0
R3	(CA)_8_T	4	3	75.0
R4	(GA)_8_YT	3	2	66.6
R5	(AG)_8_T	2	1	50.0
R6	(AG)_8_C	1	0	0
R7	(CA)_8_G	3	2	66.6
R8	(GGAGA)_3_	3	2	66.6
R9	(CA)_8_RC	5	3	60.0
R10	(GA)_8_C	4	2	50.0
R11	(GA)_7_C	3	2	66.6
R12	(AG)_8_YA	1	0	0
R13	(AG)_8_YT	7	6	85.7
R14	(AG)_9_YC	5	4	80.0
R15	(GAA)_5_	3	2	66.6
R16	(AC)_8_G	1	0	0
R17	(AC)_8_T	10	9	90.0
R18	(GA)_8_T	3	2	66.6
R19	(GATA)_2_(GACA)_2_	5	4	80.0
R20	(CTC)_6_	1	0	0
R21	(GA)_8_A	4	3	75.0
R22	(CA)_8_C	2	1	50.0
R23	(GA)_8_G	1	0	0
R24	(TAC)_8_G	0	0	0
Total		76	52	
Mean		3.1	2.1	68.4

**Table 2:** DNA GC content of the genus *Pleurotus.*

Taxa	GC content (mol%)	
	Previous data	This study
*P. abalonus*	—	47.1 ± 1.8
*P. abieticola*	44.49^[58]^	47.0 ± 0.9
*P. australis*	—	49.3 ± 0.6
*P. calyptratus*	—	44.5 ± 0.4
*P. citrinopileatus*	49.2^[59]^, 44.8^[58]^	49.0 ± 1.6
*P. columbinus*	—	46.8 ± 1.3
*P. cornucopiae*	—	50.4 ± 0.5
*P. cystidiosus*	—	47.3 ± 1.6
*P. djamor*	—	48.3 ± 1.8
*P. dryinus*	—	49.5 ± 1.2
*P. elongatipes*	—	46.5 ± 0.8
*P. eous*	—	50.0 ± 1.9
*P. eryngii*	49.1^[60]^, 49.4^[59]^, 49.3^[61]^	49.4 ± 0.9
*P. euosmus*	—	51.7 ± 1.1
*P. eryngii* var. *ferulae*	49.9^[61]^	50.4 ± 0.7
*P. flabellatus*	—	50.3 ± 0.7
*P. ostreatus* ^∗^	—	48.8 ± 0.8
*P. ostreatus* ^∗∗^	50.9^[61]^	48.8 ± 1.9
*P. fossulatus*	—	48.0 ± 0.5
*P. fuscus*	—	50.9 ± 1.1
*P. fuscus* var*. ferulae*	—	55.6 ± 0.6
*P. incarnatus*	—	47.8 ± 1.6
*P. nebrodensis*	—	44.5 ± 0.5
*P. opuntiae*	—	43.3 ± 0.9
*P. ostreatoroseus*	52.6^[12]^	47.0 ± 1.9
*P. ostreatus*	50.4^[60]^, 50.9^[59]^, 50.8^[62]^	47.4 ± 1.9
*P. populinus*	44.9 [58]	50.3 ± 1.1
*P. pulmonarius*	51.0^[53]^, 44.5^[58]^	50.3 ± 1.3
*P. purpureo-olivaceus*	—	47.7 ± 0.8
*P. rattenburyi*	—	52.4 ± 1.0
*P. salmoneostramineus*	50.0^[59]^	49.1 ± 0.9
*P. sapidus*	44.9^[58]^	51.8 ± 1.5
*P. smithii*	—	45.1 ± 0.5
*P. spodoleucus*	—	46.6 ± 0.6
*P. subareolatus*	—	47.9 ± 0.8
*P. tuber-regium*	46.1^[59]^	46.1 ± 0.5

^∗^ *P. ostreatus* was also named as *P. florida*, including strains P144, P126, P123, P130, P138, P114, P107, P119, and P128. ^∗∗^*P. ostreatus* was also named as *P. floridanus*, including strains P108, P145, P111, and P152. —: no data.

**Table 3:** Chemical characteristics of the genus *Pleurotus.*

Taxa	Sugar	Amino acid	Polar lipid
*P. abalonus*	Gal, Glu, Ara, Xyl, Rib	Asp, Arg, Lys, Thr, Glc, Tyr, Ala, Val	DPG, PME, PC, PE, PG, PS, GL, L
*P. abieticola*	Gal, Glu, Ara, Xyl, Rib, Rha	Asp, Gly, Arg, Lys, Thr, Glc, Tyr, Ala, Val	DPG, PME, PC, PE, PG, PS, GL, L
*P. australis*	Glu, Ara, Xyl, Rib	Asp, Arg, Lys, Thr, Glc, Tyr, Ala, Val	DPG, PME, PC, PE, PG, PS, GL, L
*P. calyptratus*	Gal, Glu, Ara, Xyl, Rib	Asp, Gly, Arg, Lys, Thr, Glc, Tyr, Ala, Val	DPG, PME, PC, PE, PG, PS, GL, L
*P. citrinopileatus*	Gal, Glu, Ara, Xyl	Asp, Gly, Arg, Lys, Thr, Glc, Tyr, Ala, Val	DPG, PME, PC, PE, PG, PS, PL, AL, GL, L
*P. columbinus*	Gal, Glu, Ara, Xyl, Rib	Asp, Gly, Arg, Lys, Glc, Tyr, Ala, Val	DPG, PME, PC, PE, PG, PS, AL, GL, L
*P. cornucopiae*	Gal, Glu, Ara, Xyl, Rib	Asp, Arg, Lys, Thr, Glc, Tyr, Ala, Val	DPG, PME, PC, PE, PG, PS, PL, GL, L
*P. cystidiosus*	Gal, Glu, Ara, Xyl, Rib	Asp, Arg, Lys, Thr, Glc, Tyr, Ala, Val	DPG, PME, PC, PE, PG, PS, GL, L
*P. djamor*	Gal, Glu, Ara, Xyl, Rib	Asp, Gly, Arg, Lys, Thr, Glc, Tyr, Ala, Val	DPG, PME, PC, PE, PG
*P. dryinus*	Gal, Glu, Ara, Xyl, Rib	Asp, Gly, Arg, Lys, Thr, Glc, Tyr, Ala, Val	DPG, PME, PC, PE, PG, PS
*P. elongatipes*	Gal, Glu, Ara, Xyl, Rib	Cys, Asp, Gly, Arg, Lys, Thr, Glc, Tyr, Ala, Val	DPG, PME, PC, GL
*P. eous*	Gal, Glu, Ara, Xyl, Rib	Asp, Gly, Arg, Lys, Glc, Tyr, Ala, Val	DPG, PME, PC, PE, PG, AL, L
*P. eryngii*	Gal, Glu, Ara, Xyl, Rib	Asp, Gly, Arg, Lys, Thr, Glc, Tyr, Ala, Val	DPG, PME, PC, PE, PG, PL, AL, GL, L
*P. euosmus*	Gal, Glu, Ara, Xyl, Rib	Asp, Gly, Arg, Lys, Thr, Glc, Tyr, Ala, Val	DPG, PME, PC, PE, PG, PL, GL
*P. ferulae*	Gal, Glu, Ara, Xyl, Rib	Asp, Gly, Arg, Lys, Thr, Glc, Tyr, Ala, Val	DPG, PME, PC, PE, PG, PL, AL, GL, L
*P. flabellatus*	Gal, Glu, Ara, Xyl, Rib	Asp, Arg, Lys, Thr, Glc, Tyr, Ala, Val	DPG, PME, PC, PE, PG, PS, GL, L
*P. ostreatus* ^∗^	Gal, Glu, Ara, Xyl, Rib	Asp, Gly, Arg, Lys, Thr, Glc, Tyr, Ala, Val	DPG, PME, PC, PE, PG, PS, PL, AL, GL, L
*P. ostreatus* ^∗∗^	Gal, Glu, Ara, Xyl, Rib	Asp, Arg, Lys, Thr, Glc, Tyr, Ala, Val	DPG, PME, PC, PE, PG, PS, GL, L
*P. fossulatus*	Gal, Glu, Ara, Xyl, Rib	Asp, Gly, Arg, Lys, Thr, Glc, Tyr, Ala, Val	DPG, PME, PC, PE, PS, L
*P. fuscus*	Gal, Glu, Ara, Xyl, Rib	Asp, Gly, Arg, Lys, Thr, Glc, Tyr, Ala, Val	DPG, PME, PC, PE, PG, PL, AL, GL, L
*P. fuscus* var*. ferulae*	Gal, Glu, Ara, Xyl, Rib	Asp, Gly, Arg, Lys, Thr, Glc, Tyr, Ala, Val	DPG, PME, PC, PE, PG, PL, AL, GL, L
*P. incarnatus*	Gal, Glu, Ara, Xyl, Rib	Asp, Gly, Arg, Lys, Thr, Glc, Tyr, Ala, Val	DPG, PME, PC, PE, PG, PL, AL, GL, L
*P. nebrodensis*	Gal, Glu, Ara, Xyl, Rib	Asp, Arg, Lys, Tyr, Ala	DPG, PME, PC, PE, PG, PL, AL, GL, L
*P. opuntiae*	Gal, Glu, Ara, Xyl, Rib	Asp, Gly, Arg, Lys, Thr, Glc, Tyr, Ala	DPG, PME, PC, PE, AL
*P. ostreatoroseus*	Gal, Glu, Ara, Xyl, Rib	Asp, Gly, Arg, Lys, Thr, Glc, Tyr, Ala, Val	DPG, PME, PC, PE, PG, PL, AL, GL, L
*P. ostreatus*	Glu, Ara, Xyl, Rib	Asp, Gly, Arg, Lys, Thr, Glc, Tyr, Ala, Val	DPG, PME, PC, PE, PG, PS, PL, AL, GL, L
*P. populinus*	Gal, Glu, Ara, Xyl, Rib	Asp, Gly, Arg, Lys, Thr, Glc, Tyr, Ala, Val	DPG, PME, PC, PE, PG, PL, L
*P. pulmonarius*	Glu, Ara, Xyl	Asp, Gly, Arg, Lys, Thr, Glc, Tyr, Ala, Val	DPG, PME, PC, PE, PG, PS, PL, AL, GL, L
*P. purpureo-olivaceus*	Gal, Glu, Ara, Xyl, Rib	Asp, Gly, Arg, Lys, Thr, Glc, Tyr, Ala, Val	DPG, PME, PC, PE, L
*P. rattenburyi*	Gal, Glu, Ara, Xyl, Rib	Asp, Gly, Arg, Lys, Thr, Glc, Tyr, Ala, Val	DPG, PME, PC, PE, PS, L
*P. salmoneostramineus*	Gal, Glu, Ara, Xyl, Rib	Asp, Gly, Arg, Lys, Thr, Glc, Tyr, Ala, Val	DPG, PME, PC, PE, PG
*P. sapidus*	Gal, Glu, Ara, Xyl, Rib	Asp, Arg, Lys, Thr, Glc, Tyr, Ala, Val	DPG, PME, PC, PE, PG, PS, PL, AL, L
*P. smithii*	Glu, Ara, Xyl	Asp, Gly, Arg, Lys, Thr, Glc, Tyr, Ala, Val	DPG, PME, PC, PE, PG, PS, GL, L
*P. spodoleucus*	Gal, Glu, Ara, Xyl, Rib	Asp, Gly, Arg, Lys, Thr, Glc, Tyr, Ala, Val	DPG, PME, PC, PE, PG, PS, L
*P. subareolatus*	Glu, Ara, Xyl, Rib, Rha	Asp, Arg, Lys, Tyr, Ala	DPG, PME, PC, PE, PG, PS, PL, AL, GL, L
*P. tuber-regium*	Gal, Glu, Ara, Xyl, Rib, Rha	Cys, Asp, Arg, Lys, Glc, Tyr, Ala, Val	PME, PC, PE, PG, PS, GL

Gal: galactose; Glu: glucose; Ara: arabinose; Xyl: xylose; Rib: ribose; Rha: rhamnose; Cys: cysteine; Asp: aspartic acid; Gly: glycine; Arg: arginine; Lys: lysine; Thr: threonine; Glc: glutamic acid; Tyr: tyrosine; Ala: alanine; Val: valine; DPG: diphosphatidylglycerol; PG: phosphatidylglycerol; PME: phosphatidyl-N-methylethanolamine; PC: phosphatidylcholine; PS: phosphatidylserine; PE: phosphatidylethanolamine; PL: unidentified phospholipid; AL: unidentified aminolipid; GL: unidentified glycolipid; L: unidentified lipid. ^∗^*P. ostreatus* was also named as *P. florida* (invalid name), including strains P144, P126, P123, P130, P138, P114, P107, P119, and P128. ^∗∗^*P. ostreatus* was also named as *P. floridanus* (invalid name), including strains P108, P145, P111, and P152.

**Table 4:** Quinone analysis of the genus *Pleurotus.*

Taxa	Quinone
Genus *Pleurotus*	MK-6
*Lentinellus ursinus* YTH 267	MK-7
*Hohenbuehelia petaloides* YTH 3549	MK-7(H2)
*Resupinatus applicatus* YTH 498	MK-8
*Phyllotopsis nidulans* YTH 5876	MK-5

*L. ursinus* YTH 267, *H. petaloides* YTH 3549, *R. applicatus* YTH 498, and *P. nidulans* YTH 5876 have been deposited in the College of Life Science, Kyung Hee University.

**(a):** 

Fatty acid	Taxa no.																		
	1	2	3	4	5	6	7	8	9	10	11	12	13	14	15	16	17	18	19
C_9:0_	—	—	—	—	—	—	—	Tr	—	—	1.2	—	—	—	5	—	—	—	—
C_10:0_	—	—	—	—	—	—	—	Tr	—	—	—	3.9	4.2	—	Tr	—	—	1.8	—
C_12:0_	—	—	—	—	—	—	—	—	—	—	—	—	—	—	4.1	—	—	—	—
C_14:0_	—	—	—	—	—	—	—	Tr	—	Tr	—	—	—	—	—	—	—	—	—
C_15:0_	—	—	—	—	—	—	—	Tr	—	Tr	—	—	—	—	2	—	—	—	—
C_16:0_	42.8	35.7	32.3	21.1	30.0	100	7.1	22.7	16.5	20.3	26.8	19.9	24.8	10.3	21.3	26.5	15.5	15.3	19.6
C_17:0_	—		—	—	—	—	—	—	—	Tr	—	—	—	—	—	—	—	—	—
C_18:0_	17.9	—	—	31.4	9.8	—	—	13,0	4.0	17.5	7.4	6.9	—	—	4	8.7	—	3.6	—
C_20:0_	—	—	—	—	—	—	—	Tr	—	—	—	—	—	—	—	—	—	—	—
C_12:0_ 2-Me	—	—	—	—	—	—	—	Tr	—	—	—	—	—	—	—	—	—	—	—
C_18:1_ 2OH	—	—	—	—	—	—	—	1.6	—	—	—	—	—	—	—	—	—	—	—
C_16:1_*ω*5*c*	—	—	—	—	—	—	—	—	—	—	—	—	—	—	1.1	—	—	—	—
C_16:1_*ω*7*c* alcohol	—	—	—	—	—	—	3.7	—	—	Tr	—	4.4	—	—	—	—	—	—	—
C_16:1_*cis*-11 (*ω*5*c*)	—	—	—	—	—	—	—	2.3	—	—	—	2.5	—	—	—	—	—	—	—
C_17:1_*cis*-10 (*ω*7*c*)	—	—	—	—	—	—	2.8	—	—	—	—	2.6	—	—	—	—	—	—	—
C_18:2_*cis*-9,12	—	20.2	21.1	—	31.3	—	—	11.1	33.4	35.5	48.0	—	17.8	10.4	28.8	24.4	37.7	27.4	36.7
C_18:3_*cis*-6,12,14	—	—	—	—	—	—	22.16	—	13.18	—	—	22.13	—	—	—	—	—	—	—
C_19:1_*trans*-7 (*ω*9*c*)	—	—	—	—	—	—	—	—	—	—	1.9	—	—	—	—	9.4	10.4	—	9.5
C_20:1_ N alcohol	—	—	—	—	—	—	—	—	—	—	2.5	—	—	—	—	—	—	—	—
C_20:1_*cis*-11 (*ω*9*c*)	—	—	—	—	—	—	—	—	—	1.5	—	—	—	—	—	—	—	—	—
*Iso*-C_11:0_ 3OH	—	—	—	—	—	—	19.8	—	—	—	—	—	—	—	—	—	—	—	—
*Iso*-C_14:1_	—	—	—	—	—	—	24	—	7.5	Tr	—	4.2	—	—	Tr	—	—	—	—
*Iso*-C_18:1_	—	—	—	—	—	—	3.7	—	—	12	—	5.9	—	—	Tr	—	—	—	—
*Iso*-C_19:0_	—	—	—	—	—	—	—	0.7	—	—	—	—	—	—	1.9	—	—	—	—
*Anteiso*-C_14:0_	39.3	21.3	24.3	29.5	12.5	—	12.6	0.4	5.3	1.3	2.6	11.8	—	21.2	2.4	18.2	15.2	12.4	22.2
*Anteiso*-C_15:0_	—	—	—	—	—	—	4.2	—	—	Tr	—	2.7	—	—	—	—	—	—	—
*Anteiso*-C_17:0_	—	—	—	—	—	—	—	—	—	Tr	—	—	—	—	2	—	—	—	—
Summed feature 2^∗^	—	—	—	—	—	—	—	Tr	—	—	—	—	—	—	—	—	—	—	—
Summed feature 4^∗^	—	—	—	—	—	—	—	—	—	—	—	—	—	45.3	Tr	—	—	—	—
Summed feature 8^∗^	—	15.0	—	—	11.5	—	—	45.6	15.2	28.6	9.7	8.2	53.2	—	22.6	12.9	13.8	39.5	12
Summed feature 12^∗^	—	—	—	—	—	—	—	—	4.8	—	—	—	—	—	—	—	7.4	—	—
Total unsaturated	0	20.2	21.1	0	31.3	0	56.3	15	54	36.9	49.9	41.6	17.8	10.4	31	33.8	48	27.3	46.1
Total saturated	100	57.0	56.6	82	52.3	0	43.6	39.3	25.8	32.9	40.3	42.5	29.0	31.5	43.5	53.3	30.7	33	41.8
Summed feature	0	15.0	0	0	11.5	0	0	45.7	20.0	28.6	9.7	8.2	53.2	45.3	23.2	12.9	21.2	39.5	12
Ratio U : S^a^	0	0.3	0.3	0	0.6	0	1.2	0.3	2	1.1	1.2	0.9	0.6	0.3	0.7	0.6	1.5	0.8	1.1

**(b):** 

Fatty acid	Taxa no.																
	20	21	22	23	24	25	26	27	28	29	30	31	32	33	34	35	36
C_9:0_	—	—	—	6.7	—	—	3.8	—	—	—	Tr	—	—	—	1.5	—	—
C_10:0_	—	2.3	—	3.8	Tr	—	1.6	—	—	—	Tr	—	4.9	8.5	—	—	—
C_12:0_	—	—	—	—	—	—	—	—	—	—	—	—	5	—	—	—	4.2
C_14:0_	—	—	—	—	Tr	—	—	—	—	—	0.6	—	—	—	—	—	—
C_15:0_	1.6	—	—	—	0.84	—	1.91	—	3.29	—	0.74	—	4.3	—	1.7	—	—
C_16:0_	28.3	34.9	41	25.1	29.9	41	29.8	25.2	45.7	27.3	13.8	27.9	9.6	14.5	35.1	7.4	34
C_18:0_	—	13.7	13.7	12.2	15.6	16.4	9.6	5.6	24.4	10.4	2.2	7.9	—	—	13	—	12.1
C_8:0_ 3OH	**—**	**—**	**—**	—	**—**	**—**	—	**—**	—	—	**—**	**—**	—	**—**	**—**	**—**	3.1
C_12:0_ 2-Me	—	—	—	—	—	—	—	—	—	—	—	—	2.5	6.3	—	—	—
C_14:0_ 2-Me	—	—	—	—	—	—	—	—	—	—	—	—	—	—	—	—	4.1
C_17:0_ 3OH	—	—	—	—	—	—	—	—	—	—	—	—	14.6	—	—	—	—
C_18:1_ 2OH	—	—	—	—	—	—	—	—	—	—	1.3	—	—	—	—	—	—
C_16:1_*ω*5*c*	2.3	—	—	—	—	—	—	—	—	—	—	—	—	—	—	—	—
C_16:1_*ω*7*c* alcohol	—	—	—	—	—	5.6	—	—	—	—	Tr	—	5.2	—	—	—	—
C_16:1_*cis*-9 (*ω*7*c*)	—	—	—	—	—	—	—	—	—	—	1.2	—	—	—	—	—	—
C_16:1_*cis*-11 (*ω*5*c*)	—	—	—	—	1.9	—	—	—	—	—	—	—	—	—	—	—	—
C_17:1_*cis*-10 (*ω*7*c*)	—	—	—	—	—	—	—	—	—	—	—	—	3.5	—	—	—	—
C_18:2_*cis*-9,12	25.3	15.7	29.3	6.2	24.8	—	32.9	18.4	9.4	7.3	—	23.2	—	12.3	10.7	15.2	—
C_18:3_*cis*-6,12,14	—	—	—	—	—	—	—	—	—	—	24.1	—	—	—	—	—	—
C_19:1_*trans*-7 (*ω*9*c*)	5.6	—	—	—	—	14	—	—	—	—	1.2	—	—	—	2.3	6.9	3.9
*Iso*-C_10:0_	—	—	—	—	—	—	—	—	—	—	Tr	—	4.4	—	—	—	—
*Iso*-C_14:1_	—	—	—	—	—	—	—	—	—	—	Tr	—	4.3	—	—	—	—
*Iso*-C_15:1_	—	—	—	—	—	—	—	—	—	—	—	—	8.9	—	—	—	—
*Iso*-C_17:1_ G (*ω*11*c*)	—	—	—	—	—	—	—	—	—	—	—	—	—	—	—	—	5.7
*Iso*-C_18:1_	—	—	—	—	—	—	—	—	—	—	Tr	—	5	—	—	—	—
*Iso*-C_19:0_	—	—	—	—	—	—	—	—	—	—	1.2	—	—	—	—	—	—
*Anteiso*-C_14:0_	—	14.9	7.9	25.9	1.8	23.0	5.4	10.8	17.2	7.4	2.3	6	16.1	31.8	—	65.5	12.6
*Anteiso*-C_15:0_	—	—	—	—	—	—	—	—	—	—	0.4	—	3.9	—	—	—	2.9
*Anteiso*-C_16:0_	—	—	—	—	—	—	—	—	—	—	—	—	3.2	—	—	—	—
*Anteiso*-C_17:0_	—	—	—	—	—	—	—	—	—	—	—	—	4.5	—	—	—	—
Summed feature 1^∗^	—	—	—	—	—	—	—	—	—	—	—	—	—	—	—	—	16.9
Summed feature 8^∗^	42.6	18.6	8.1	8.7	23.9	—	15	40.1	—	43.2	48.2	35.0	—	26.7	35.1	—	—
Summed feature 12^∗^	—	—	—	11.4	—	—	—	—	—	—	—	—	—	—	—	—	—
Total unsaturated	33.2	15.6	29.3	6.2	26.7	19.5	32.9	18.4	9.4	7.3	29.3	23.1	27	12.3	12.9	27	9.5
Total saturated	29.9	65.7	62.6	73.7	49.3	80.4	52	41.5	90.5	45.1	22.4	41.7	68.7	61	51.2	72.9	66.4
Summed feature	42.6	18.5	8.1	20.1	23.9	0	15	40.1	0	43.2	48.2	35	0	26.7	35.1	0	16.9
Ratio U : S^a^	1.1	0.2	0.4	0.08	0.5	0.2	0.6	0.4	0.1	0.1	1.3	0.5	0.3	0.2	0.2	0.3	0.1

Taxa no.: 1—*P. abalonus*; 2—*P. abieticola*; 3—*P. australis*; 4—*P. calyptratus*; 5—*P. citrinopileatus*; 6—*P. columbinus*; 7—*P. cornucopiae*; 8—*P. cystidiosus*; 9—*P. djamor*; 10—*P. dryinus*; 11—*P. elongatipes*; 12—*P. eous*; 13—*P. eryngii*; 14—*P. euosmus*; 15—*P. eryngii* var. *ferulae*; 16—*P. flabellatus*; 17—*P. ostreatus*^∗^ (*P. florida*); 18—*P. ostreatus*^∗∗^ (*P. floridanus*); 19—*P. fossulatus*; 20—*P. fuscus*; 21—*P. fuscus* var. *ferulae*; 22—*P. incarnatus*; 23—*P. nebrodensis*; 24—*P. opuntiae*; 25—*P. ostreatoroseus*; 26—*P. ostreatus*; 27—*P. populinus*; 28—*P. pulmonarius*; 29—*P. purpureo-olivaceus*; 30—*P. rattenburyi*; 31—*P. salmoneostramineus*; 32—*P. sapidus*; 33*—P. smithii*; 34—*P. spodoleucus*; 35—*P. subareolatus*; 36—*P. tuber-regium*. Fatty acid profiles were presented in percent area (% of total area). —: not detected. Tr: small amounts (less than 1%) are not shown. ^∗^Summed features are groups of two or three fatty acids that cannot be separated by GLC with the MIDI system. Summed feature 1 comprised C_14: l_*ω*5*c*, C_14: l_*ω*5*t*, or both. Summed feature 8 comprised C_18:1_*ω*7*c* and/or C_18:1_*ω*6*c*; summed feature 2 comprised C_14:0_ 3OH and/or *iso*-C_16:1_ I. Summed feature 8 comprised C_18:1_*ω*7*c* and/or C_18:1_*ω*6*c*. Summed feature 12 comprised *iso*-C_17:1_*ω*9*c* and/or C_16:0_ 10-Me. Summed feature 4 comprised C_16:1_*ω*7*c*/*iso*-C_15:0_. ^a^Ratio of unsaturated : saturated fatty acids. Fatty acid profiles are presented in percent area (% of total area). For the same species, values on the same row followed by the same letter are not significantly different according to the least squares means (LS means) test (*P* < 0.05).

## Data Availability

The raw data of ITS and LSU have been submitted to NCBI.

## Supplementary Materials

Supplementary Materials Supplementary Table S1: a list of species and GenBank accession number for sequences used in this study. Supplementary Table S2: a list of reference sequences from GenBank. Supplementary Figure S1: the conserved domains in the genus *Pleurotus* and primer locations. Supplementary Figure S2: optimal primer selection for ISSR analysis. Supplementary Figure S3: optimal annealing temperature and number of cycle selection for ISSR analysis (Supplementary Materials).
