# Supplementary material for: Genetic and Chemical Diversity of Edible Mushroom Pleurotus Species
Source: Biomed Res Int. 2022 Jan 15;2022:6068185. doi: 10.1155/2022/6068185 (PMC8783721; doi:10.1155/2022/6068185)
Supplement: Supplementary Materials — Supplementary Table S1: a list of species and GenBank accession number for sequences used in this study. Supplementary Table S2: a list of reference sequences from GenBank. Supplementary Figure S1: the conserved domains in the genus Pleurotus and primer locations. Supplementary Figure S2: optimal primer selection for ISSR analysis. Supplementary Figure S3: optimal annealing temperature and number of cycle selection for ISSR analysis (Supplementary Materials). [file 6068185.f1.docx]

**Supplementary materials**

**Genetic and chemical diversity of edible *mushroom Pleurotus species***

Pei Lin^1,2^, Zheng-Fei Yan^3*^, MooChang Kook^4^, Chang-Tian Li^1^, and Tae-Hoo Yi^5^

^1^ Engineering Research Center of Edible and Medicinal Fungi, Ministry of Education, Jilin Agricultural University, Changchun 130118, Jilin Province, China

^2^ School of Pharmaceutical Sciences, Jiangnan University, 1800 Lihu 12 Avenue, Wuxi 21422, Jiangsu Province, China

^3^ State Key Laboratory of Food Science and Technology, Jiangnan University, 1800 Lihu Avenue, Wuxi 21422, Jiangsu Province, China

^4^ Department of Food & Nutrition, Baewha Women’s University, Seoul, Republic of Korea

^5^ College of Life Science, Kyung Hee University, Yongin-si, Gyeonggi-do 17104, Republic of Korea

Corresponding Author:

Zheng-Fei Yan

Jiangnan University, 1800 Lihu Avenue, Wuxi 21422, Jiangsu Province, China

Tel and Fax: 86-0510-85326653

E-mail: zhengfeiyan@jiangnan.edu.cn

**Figure S1** The conserved domains in genus *Pleurotus* and primer locations


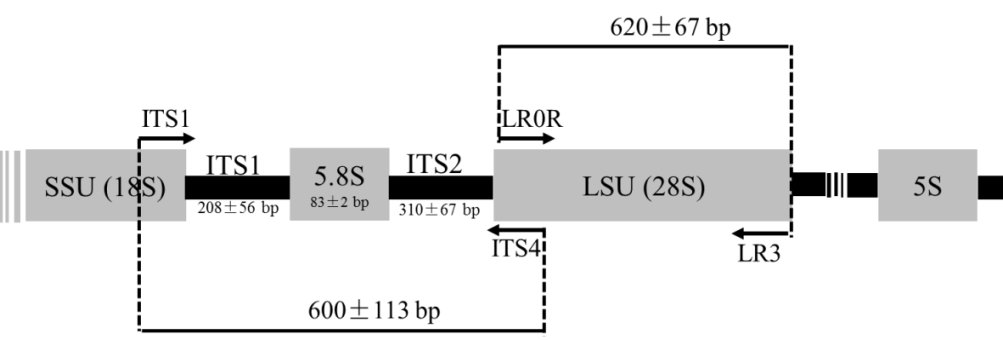


| **Domains** | **Primer name** | **Sequence** | **Reference** **PMID^#^** |
| --- | --- | --- | --- |
| ITS | ITS1 (primer forward): | 5’-TCCGTAGGTGAACCTGCGG-3’ | 23983530 |
|  | ITS4 (primer reverse): | 5’-TCCTCCGCTTATTGATATGC-3’ |  |
|  |  |  |  |
| LUS | LR0R (primer forward): | 5’-ACCCGCTGAACTTAAGC-3’ | 28154480 |
|  | LR3 (primer reverse): | 5’-CCGTGTTTCAAGACGGG-3’ |  |

Three conserved regions in genus *Pleurotus*: SSU (18S), ITS and LSU (28S). The ITS gene was located at between SSU and LSU genes, which contain three domains: ITS2, 5.8S and ITS1. Full sequences of ITS gene were amplified by pirmers ITS1 and ITS4, which have 600±113 bp. Full sequences of LSU were amplified by pirmers LR0R and LR3, which have 620±67 bp.

PMID^#^: PubMed identifier (PMID) is a unique number assigned to each PubMed record.

**Figure S2** Optimal primer selection for ISSR analysis

M, DL 1000 genetic size marker; R1-R24, various primers (detail in Table 1)

**Figure S3** Optimal annealing temperature and number of cycles selection for ISSR analysis

A: Annealing temperature (℃), M, 1000 genetic size marker; 1, 47℃; 2, 50℃; 3, 53℃; 4, 60℃; 5, 65℃ and 6, 70℃. B: Number of cycles, M, 1000 genetic size marker; 1, 10 cycles; 2, 15 cycles; 3, 20 cycles; 4, 25 cycles; 5, 30 cycles and 6, 35 cycles.

**Table S1** A list of species and GenBank accession number for sequences used in this study

| Taxa | Sample no. | MycoBank no. | GenBank no. | | Culture Collection | |
| --- | --- | --- | --- | --- | --- | --- |
|  |  |  | ITS | LSU | Name | No. |
| *P. abalonus* | P133 | 320718 | MG282472 | MG282532 | *P. abalonus* | KACC 53755 |
| *P. abalonus* | P25 |  | KY962441 | KY963025 | *P. abalonus* | NAAS 00596 |
|  |  |  |  |  |  |  |
| *P. abieticola* | P97 | 436464 | MG282437 | MG282496 | *P. abieticola* | KACC 46261 |
|  |  |  |  |  |  |  |
| *P. australis* | P49 | 198080 | KY962465 | KY963049 | *P. australis* | KACC 52411 |
| *P. australis* | P66 |  | KY962482 | KY963066 | *P. australis* | NAAS 00676 |
|  |  |  |  |  |  |  |
|  |  |  |  |  |  |  |
| *P. calyptratus* | P67 | 233195 | KY962483 | KY963067 | *P. calyptratus* | NAAS 00677 |
| *P. calyptratus* | P71 |  | KY962487 | KY963071 | *P. calyptratus* | NAAS 00744 |
|  |  |  |  |  |  |  |
| *P. citrinopileatus* | P18 | 303973 | KY962434 | KY963018 | *P. citrinopileatus* | CCMJ 3251 |
| *P. citrinopileatus* | P28 |  | KY962444 | KY963028 | *P. citrinopileatus* | KACC 42707 |
| *P. citrinopileatus* | P38 |  | KY962454 | KY963038 | *P. citrinopileatus* | KACC 42734 |
| *P. citrinopileatus* | P68 |  | KY962484 | KY963068 | *P. citrinopileatus* | NAAS 00710 |
| *P. citrinopileatus* | P81 |  | KY962497 | KY963081 | *P. citrinopileatus* | NAAS 02270 |
| *P. citrinopileatus* | P10 |  | KY962426 | KY963010 | *P. citrinopileatus* | KACC 46267 |
|  |  |  |  |  |  |  |
| *P. columbinus* | P118 | 159901 | MG282457 | MG282517 | *P. columbinus* | KACC 51432 |
| *P. columbinus* | P125 |  | MG282464 | MG282524 | *P. columbinus* | KACC 52299 |
| *P. columbinus* | P137 |  | MG282476 | MG282536 | *P. columbinus* | KACC 54315 |
| *P. columbinus* | P143 |  | MG282482 | MG282542 | *P. columbinus* | KACC 54354 |
| *P. columbinus* | P96 |  | MG282436 | MG282495 | *P. columbinus* | KACC 42745 |
|  |  |  |  |  |  |  |
| *P. cornucopiae* | P16 | 355897 | KY962432 | KY963016 | *P. cornucopiae* | CCMJ 3548 |
| *P. cornucopiae* | P42 |  | KY962458 | KY963042 | *P. cornucopiae* | KACC 46269 |
| *P. cornucopiae* | P54 |  | KY962470 | KY963054 | *P. cornucopiae* | KACC 54252 |
| *P. cornucopiae* | P69 |  | KY962485 | KY963069 | *P. cornucopiae* | NAAS 00711 |
| *P. cornucopiae* | P88 |  | KY962504 | KY963088 | *P. cornucopiae* | NAAS 04559 |
|  |  |  |  |  |  |  |
| *P. cystidiosus* | P43 | 337245 | KY962459 | KY963043 | *P. cystidiosus* | KACC 46270 |
| *P. cystidiosus* | P89 |  | KY962505 | KY963089 | *P. cystidiosus* | NAAS 04662 |
| *P. cystidiosus* | P77 |  | KY962493 | KY963077 | *P. cystidiosus* | NAAS 00830 |
| *P. cystidiosus* | P86 |  | KY962502 | KY963086 | *P. cystidiosus* | NAAS 04367 |
|  |  |  |  |  |  |  |
| *P. djamor* | P29 | 355683 | KY962445 | KY963029 | *P. djamor* | KACC 42708 |
| *P. djamor* | P61 |  | KY962477 | KY963061 | *P. djamor* | NAAS 00219 |
| *P. djamor* | P91 |  | KY962507 | KY963091 | *P. djamor* | NAAS 06004 |
|  |  |  |  |  |  |  |
| *P. dryinus* | P45 | 151965 | KY962461 | KY963045 | *P. dryinus* | KACC 46325 |
| *P. dryinus* | P99 |  | MG282439 | MG282498 | *P. dryinus* | KACC 46274 |
|  |  |  |  |  |  |  |
| *P. elongatipes* | P113 | 170687 | MG282452 | MG282512 | *P. elongatipes* | KACC 51371 |
| *P. elongatipes* | P75 |  | KY962491 | KY963075 | *P. elongatipes* | NAAS 00768 |
|  |  |  |  |  |  |  |
| *P. eous* | P109 | 492857 | MG282448 | MG282508 | *P. eous* | KACC 51357 |
| *P. eous* | P139 |  | MG282478 | MG282538 | *P. eous* | KACC 54331 |
| *P. eous* | P149 |  | MG282488 | MG282548 | *P. eous* | KACC 54404 |
| *P. eous* | P95 |  | MG282435 | MG282494 | *P. eous* | KACC 42726 |
|  |  |  |  |  |  |  |
| *P. eryngii* | P84 | 170772 | KY962500 | KY963084 | *P. eryngii* | NAAS 02677 |
| *P. eryngii* | P20 |  | KY962436 | KY963020 | *P. eryngii* | CCMJ 3459 |
| *P. eryngii* | P32 |  | KY962448 | KY963032 | *P. eryngii* | KACC 42720 |
|  |  |  |  |  |  |  |
| *P. euosmus* | P100 | 170637 | MG282440 | MG282499 | *P. euosmus* | KACC 46275 |
| *P. euosmus* | P147 |  | MG282486 | MG282546 | *P. euosmus* | KACC 54390 |
|  |  |  |  |  |  |  |
| *P. flabellatus* | P30 | 164548 | KY962446 | KY963030 | *P. flabellatus* | KACC 42709 |
| *P. flabellatus* | P35 |  | KY962451 | KY963035 | *P. flabellatus* | KACC 42723 |
| *P. flabellatus* | P65 |  | KY962481 | KY963065 | *P. flabellatus* | NAAS 00598 |
| *P. flabellatus* | P76 |  | KY962492 | KY963076 | *P. flabellatus* | NAAS 00774 |
|  |  |  |  |  |  |  |
| *P. ostreatus* | P107 | 174220 | MG282492 | MG282506 | *P. florida* | KACC 49470 |
| *P. ostreatus* | P114 |  | MG282453 | MG282513 | *P. florida* | KACC 51372 |
| *P. ostreatus* | P119 |  | MG282458 | MG282518 | *P. florida* | KACC 51433 |
| *P. ostreatus* | P123 |  | MG282462 | MG282522 | *P. florida* | KACC 52110 |
| *P. ostreatus* | P126 |  | MG282465 | MG282525 | *P. florida* | KACC 52324 |
| *P. ostreatus* | P128 |  | MG282467 | MG282527 | *P. florida* | KACC 53322 |
| *P. ostreatus* | P130 |  | MG282469 | MG282529 | *P. florida* | KACC 53439 |
| *P. ostreatus* | P138 |  | MG282477 | MG282537 | *P. florida* | KACC 54327 |
| *P. ostreatus* | P144 |  | MG282483 | MG282543 | *P. florida* | KACC 54356 |
| *P. ostreatus* | P108 |  | MG282447 | MG282507 | *P. floridanus* | KACC 51352 |
| *P. ostreatus* | P111 |  | MG282450 | MG282510 | *P. floridanus* | KACC 51365 |
| *P. ostreatus* | P145 |  | MG282484 | MG282544 | *P. floridanus* | KACC 54357 |
| *P. ostreatus* | P152 |  | MG282491 | MG282551 | *P. floridanus* | KACC 54533 |
| *P. ostreatus* | P1 |  | KY962417 | KY963001 | *P. ostreatus* | KACC 42738 |
| *P. ostreatus* | P2 |  | KY962418 | KY963002 | *P. ostreatus* | KACC 42739 |
| *P. ostreatus* | P4 |  | KY962420 | KY963004 | *P. ostreatus* | KACC 50356 |
| *P. ostreatus* | P5 |  | KY962421 | KY963005 | *P. ostreatus* | KACC 53073 |
| *P. ostreatus* | P6 |  | KY962422 | KY963006 | *P. ostreatus* | Market^1^ |
| *P. ostreatus* | P7 |  | KY962423 | KY963007 | *P. ostreatus* | Market^2^ |
| *P. ostreatus* | P8 |  | KY962424 | KY963008 | *P. ostreatus* | Market^3^ |
| *P. ostreatus* | P9 |  | KY962425 | KY963009 | *P. ostreatus* | Market^4^ |
| *P. ostreatus* | P17 |  | KY962433 | KY963017 | *P. ostreatus* | CCMJ 3069 |
| *P. ostreatus* | P24 |  | KY962440 | KY963024 | *P. ostreatus* | KCCM 60165 |
| *P. ostreatus* | P26 |  | KY962442 | KY963026 | *P. ostreatus* | KACC 42305 |
| *P. ostreatus* | P48 |  | KY962464 | KY963048 | *P. ostreatus* | KACC 49470 |
| *P. ostreatus* | P58 |  | KY962474 | KY963058 | *P. ostreatus* | NAAS 00065 |
| *P. ostreatus* | P62 |  | KY962478 | KY963062 | *P. ostreatus* | NAAS 00411 |
| *P. ostreatus* | P64 |  | KY962480 | KY963064 | *P. ostreatus* | NAAS 00597 |
| *P. ostreatus* | P72 |  | KY962488 | KY963072 | *P. ostreatus* | NAAS 00751 |
| *P. ostreatus* | P82 |  | KY962498 | KY963082 | *P. ostreatus* | NAAS 02541 |
| *P. ostreatus* | P92 |  | KY962508 | KY963092 | *P. ostreatus* | NAAS 06134 |
| *P. ostreatus* | P93 |  | KY962509 | KY963093 | *P. ostreatus* | NAAS 06421 |
|  |  |  |  |  |  |  |
| *P.* *eryngii var. ferulae* | P140 | 171113 | MG282479 | MG282539 | *P.* *eryngii var. ferulae* | KACC 54334 |
| *P.* *eryngii var. ferulae* | P21 |  | KY962437 | KY963021 | *P.* *eryngii var. ferulae* | CCMJ 4021 |
|  |  |  |  |  |  |  |
| *P. fossulatus* | P129 | 175019 | MG282468 | MG282528 | *P. fossulatus* | KACC 53383 |
| *P. fossulatus* | P87 |  | MG282485 | MG282545 | *P. fossulatus* | NAAS 04393 |
| *P. fossulatus* | P146 |  | KY962503 | KY963087 | *P. fossulatus* | KACC 54365 |
|  |  |  |  |  |  |  |
| *P. fuscus* | P150 | 265693 | MG282489 | MG282549 | *P. fuscus* | KACC 54405 |
|  |  |  |  |  |  |  |
| *P. fuscus var. ferulae* | P120 | 446047 | MG282459 | MG282519 | *P. fuscus var. ferulae* | KACC 51434 |
|  |  |  |  |  |  |  |
| *P. incarnatus* | P110 | 446050 | MG282449 | MG282509 | *P. incarnatus* | KACC 51358 |
| *P. incarnatus* | P115 |  | MG282454 | MG282514 | *P. incarnatus* | KACC 51373 |
| *P. incarnatus* | P116 |  | MG282455 | MG282515 | *P. incarnatus* | KACC 51374 |
| *P. incarnatus* | P142 |  | MG282481 | MG282541 | *P. incarnatus* | KACC 54341 |
|  |  |  |  |  |  |  |
| *P. nebrodensis* | P148 | 168802 | MG282487 | MG282547 | *P. nebrodensis* | KACC 54401 |
| *P. nebrodensis* | P151 |  | MG282490 | MG282550 | *P. nebrodensis* | KACC 54429 |
|  |  |  |  |  |  |  |
| *P. opuntiae* | P101 | 174396 | MG282441 | MG282500 | *P. opuntiae* | KACC 46276 |
| *P. opuntiae* | P124 |  | MG282463 | MG282523 | *P. opuntiae* | KACC 52287 |
| *P. opuntiae* | P132 |  | MG282471 | MG282531 | *P. opuntiae* | KACC 53511 |
|  |  |  |  |  |  |  |
| *P. ostreatoroseus* | P94 | 337253 | MG282434 | MG282493 | *P. ostreatoroseus* | KACC 42708 |
|  |  |  |  |  |  |  |
| *P. populinus* | P12 | 361394 | KY962428 | KY963012 | *P. populinus* | KACC 51352 |
| *P. populinus* | P70 |  | KY962486 | KY963070 | *P. populinus* | NAAS 00718 |
| *P. populinus* | P83 |  | KY962499 | KY963083 | *P. populinus* | NAAS 02574 |
|  |  |  |  |  |  |  |
| *P. purpureo-olivaceus* | P102 | 413855 | MG282442 | MG282501 | *P. purpureo-olivaceus* | KACC 46279 |
|  |  |  |  |  |  |  |
| *P. pulmonarius* | P14 | 143543 | KY962430 | KY963014 | *P. pulmonarius* | KACC 52410 |
| *P. pulmonarius* | P27 |  | KY962443 | KY963027 | *P. pulmonarius* | KACC 42706 |
| *P. pulmonarius* | P39 |  | KY962455 | KY963039 | *P. pulmonarius* | KACC 42705 |
| *P. pulmonarius* | P41 |  | KY962457 | KY963041 | *P. pulmonarius* | KACC 44186 |
| *P. pulmonarius* | P50 |  | KY962466 | KY963050 | *P. pulmonarius* | KACC 53660 |
| *P. pulmonarius* | P51 |  | KY962467 | KY963051 | *P. pulmonarius* | KACC 54195 |
| *P. pulmonarius* | P52 |  | KY962468 | KY963052 | *P. pulmonarius* | KACC 54202 |
| *P. pulmonarius* | P53 |  | KY962469 | KY963053 | *P. pulmonarius* | KACC 54237 |
|  |  |  |  |  |  |  |
| *P. rattenburyi* | P103 | 129740 | MG282443 | MG282502 | *P. rattenburyi* | KACC 46280 |
| *P. rattenburyi* | P112 |  | MG282451 | MG282511 | *P. rattenburyi* | KACC 51369 |
| *P. rattenburyi* | P74 |  | KY962490 | KY963074 | *P. rattenburyi* | NAAS 00757 |
|  |  |  |  |  |  |  |
| *P. salmoneostramineus* | P15 | 320725 | KY962431 | KY963015 | *P. salmoneostramineus* | CCMJ 4398 |
| *P. salmoneostramineus* | P36 |  | KY962452 | KY963036 | *P. salmoneostramineus* | KACC 42731 |
| *P. salmoneostramineus* | P60 |  | KY962476 | KY963060 | *P. salmoneostramineus* | NAAS 00117 |
|  |  |  |  |  |  |  |
| *P. sapidus* | P63 | 243659 | KY962479 | KY963063 | *P. sapidus* | NAAS 00571 |
| *P. sapidus* | P3 |  | KY962419 | KY963003 | *P. sapidus* | KACC 42744 |
| *P. sapidus* | P11 |  | KY962427 | KY963011 | *P. sapidus* | KACC 53322 |
| *P. sapidus* | P13 |  | KY962429 | KY963013 | *P. sapidus* | KACC 51359 |
| *P. sapidus* | P19 |  | KY962435 | KY963019 | *P. sapidus* | CCMJ 3609 |
| *P. sapidus* | P31 |  | KY962447 | KY963031 | *P. sapidus* | KACC 42719 |
| *P. sapidus* | P33 |  | KY962449 | KY963033 | *P. sapidus* | KACC 42721 |
|  |  |  |  |  |  |  |
| *P. smithii* | P104 | 320726 | MG282444 | MG282503 | *P. smithii* | KACC 46282 |
| *P. smithii* | P105 |  | MG282445 | MG282504 | *P. smithii* | KACC 46283 |
| *P. smithii* | P44 |  | KY962460 | KY963044 | *P. smithii* | KACC 46283 |
| *P. smithii* | P90 |  | KY962506 | KY963090 | *P. smithii* | NAAS 04682 |
|  |  |  |  |  |  |  |
| *P. spodoleucus* | P57 | 143366 | KY962473 | KY963057 | *P. spodoleucus* | NAAS 00062 |
| *P. spodoleucus* | P59 |  | KY962475 | KY963059 | *P. spodoleucus* | NAAS 00106 |
| *P. spodoleucus* | P80 |  | KY962496 | KY963080 | *P. spodoleucus* | NAAS 02099 |
|  |  |  |  |  |  |  |
| *P. subareolatus* | P106 | 246643 | MG282446 | MG282505 | *P. subareolatus* | KACC 46324 |
| *P. subareolatus* | P127 |  | MG282466 | MG282526 | *P. subareolatus* | KACC 52338 |
| *P. subareolatus* | P131 |  | MG282470 | MG282530 | *P. subareolatus* | KACC 53510 |
|  |  |  |  |  |  |  |
| *P. tuber-regium* | P141 | 303985 | MG282480 | MG282540 | *P. tuber-regium* | KACC 54335 |

KACC: Korean Agricultural Culture Collection (Kroea). NAAS: National Agrobiodiversity Center (Kroea). KCCM: Korean Culture Center of Microorganisms (Kroea). CCMJ: Culture Center of Microorganisms Jilin Agricultural University (China). Market: Strain isolated from fruit bodies of different manufacturers, which collected from Homeplus in Yongtong si of Korea

**Table S2** A list of reference sequences from GenBank

| Taxa | GenBank no. | | Reference |
| --- | --- | --- | --- |
|  | ITS | LSU |  |
| *P. abalonus* | EU365635 | EU424278 | Gao *et al* (2008) |
| *P. abieticola* | EU365634 | EU424277 | Gao *et al* (2008) |
| *P. australis* | EU365633 | EU424275 | Gao *et al* (2008) |
| *P. calyptratus* | AY562496 | AY562495 | Li *et al* (2005) |
| *P. citrinopileatus* | EU365642 | EU424285 | - |
| *P. columbinus* | EU365643 | EU424286 | - |
| *P. cornucopiae* | AY450341 | AY450341 | Li *et al* (2005) |
| *P. cystidiosus* | EU365636 | EU424279 | Gao *et al* (2008) |
| *P. djamor* | EU365644 | EU424287 | Gao *et al* (2008) |
| *P. dryinus* | AY450343 | AY450343 | Li *et al* (2005) |
| *P. elongatipes* | - | - | - |
| *P. eous* | KY214257 | KY214257 | - |
| *P. eryngii* | HM998833 | HM998797 | Zervakis *et al* (2014) |
| *P. euosmus* | EU365654 | EU424298 | Gao *et al* (2008) |
| *P. flabellatus* | EU365658 | EU424303 | Gao *et al* (2008) |
| *P. florida* | MT7870151  .1 | MT78555111  1 | - |
| *P. floridanus* | EU365656 | EU424301 | Gao *et al* (2008) |
| *P. ostreatus* | AY450345 | AY450345 | Li *et al* (2005) |
| *P.* *eryngii var. ferulae* | HM998813 | HM998778 | Zervakis *et al* (2014) |
| *P. fossulatus* | HM998828 | HM998792 | Zervakis *et al* (2014) |
| *P. fuscus* | MH443283 | MH443283 | - |
| *P. fuscus var. ferulae* | EU365655 | EU424299 | Gao *et al* (2008) |
| *P. incarnatus* | EU365661 | EU424306 | Li *et al* (2005) |
| *P. nebrodensis* | HM998835 | HM998799 | Zervakis *et al* (2014) |
| *P. opuntiae* | AY450339 | AY450339 | Li *et al* (2005) |
| *P. ostreatoroseus* | MH915573 | MH915573 | - |
| *P. populinus* | AY368667 | AY368667 | Li *et al* (2005) |
| *P. purpureo-olivaceus* | AF135179 | MH396004 | - |
| *P. pulmonarius* | EU365666 | EU424311 | Gao *et al* (2008) |
| *P. rattenburyi* | EU365670 | EU424315 | Gao *et al* (2008) |
| *P. salmoneostramineus* | EU365674 | EU424318 | Gao *et al* (2008) |
| *P. sapidus* | EU365672 | EU424316 | Gao *et al* (2008) |
| *P. smithii* | U04150 | U04084 | Zervakis *et al* (2014) |
| *P. spodoleucus* | AY265848 | AY265848 | Li *et al* (2005) |
| *P. subareolatus* | AY265849 | AY265849 | Li *et al* (2005) |
| *P. tuber-regium* | EU365676 | EU424319 | Gao *et al* (2008) |
| *Agaricus bisporus* | DQ0717710 | AJ409229 | Gao *et al* (2008) |

-: No data

Reference from **GI Zervakis *et al*.** A reappraisal of the *Pleurotus eryngii* complex-New species and taxonomic combinations based on the application of a polyphasic approach, and an identification key to Pleurotus taxa associated with Apiaceae plants. Fungal Biology. 2014 118(9-10): 814-34. **Xueling, Li *et al*.** Phylogenetic position of *Pleurotus calyptratus* based on nrDNA-LSU and ITS sequences. Journal of Beijing Forestry University. 2005 03: 67-71. **Shan,** **Gao *et al*.** Phylogenetic relationship of *Pleurotus* species based on nuclear large subunit ribosomal DNA sequences. Journal of Plant Genetic Resources. 2008 9(3): 328-334.
